# Spider-Like Coronary Anatomy; the True Spider!

**DOI:** 10.5935/abc.20170101

**Published:** 2017-10

**Authors:** Levent Cerit, Hamza Duygu, Kamil Gülşen, Hatice Kemal, Barcin Ozcem

**Affiliations:** 1 Near East University - Department of Cardiology, Nicosia, Cyprus; 2 Near East University - Department of Cardiovascular Surgery, Nicosia, Cyprus

**Keywords:** Coronary Vessels / anatomy & histology, Coronary Vessels Anomalies, Tomography, X-Ray Computed

## Introduction

Coronary anomalies are uncommon, affecting approximately 0.3-5.6% of the general population according to the literature. However, some cases can result in severe life-threatening events, such as myocardial ischemia, arrhythmia, and acute myocardial infarction.^1,2^

## Case Report

**Figure 1:**
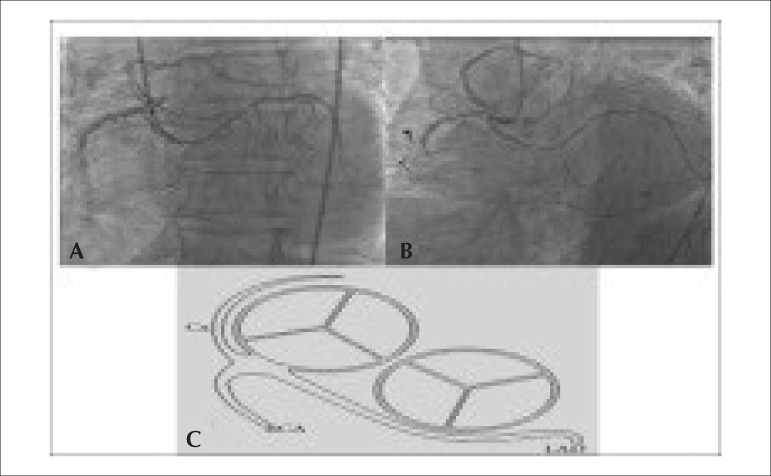
(A,B) Coronary angiographic imaging in the left anterior oblique projection showing three coronary arteries originating from right sinus of Valsalva. (C) Schematic drawing of the coronary anomaly.

A 55 year-old man was admitted to the hospital with typical chest pain; the ECG and cardiac biomarkers were normal. He had a history of hypertension and coronary angiography (CAG) a year ago due to unstable angina pectoris and a drug-eluting stent was implanted at the left anterior descending (LAD) coronary artery. CAG was performed at admission due to persistent chest pain and a single right coronary ostium was seen at the right coronary sinus, where LAD artery, left circumflex coronary (LCx) artery and right coronary artery (RCA) arose altogether. Non-significant plaques were seen at LAD and LCx, whereas RCA was obstructed from the middle segment and retrograde perfusion was observed (Figure 1A and 1B). The patient was treated conservatively and reported no chest pain 12 months later. The single coronary ostium is classified into 20 categories based on the ostium's location and our patient had characteristics of type IID^3^ (Figure 1C). Although type IID coronary anomaly has been described before, it has been reported only once and this is the second case of literature showing a single coronary ostium originating from the right coronary ostium.

## Discussion

Single coronary artery (SCA) from the right sinus of Valsalva was detected in 0.019% on coronary angiography^1^. Shirani and Roberts^2^ reported 97 cases of SCA, 51 of which originated from the right sinus of Valsalva.

Lipton et al.^3^ recommended a classification, which was modified by Yamanaka and Hobbs.^1^ Depending on the sinus of origin, the anomalous artery is designated as R (right) or L (left). It is further classified as: Type I: normal course of left or RCA with a continuation into the absent artery's territory. Type II: Anomalous artery arises from the proximal part of the other normal artery and courses the base of the heart before taking the native course. Type III: The LAD and LCx arteries arise from the proximal part of the RCA. Type III anomalies are very rare. Single coronary ostium is classified into 20 categories, based on the ostium's location and our patient had characteristics of type IID.^3^ Although type IID coronary anomaly has been described before, it has been reported only once and this is the second case in the literature showing single coronary ostium originating from the right coronary ostium.

CT angiography might be very useful in detecting the anatomical malformations, acute angle take-off, the transmural course, and compression between the great arteries, which would require surgery.^4^ Canbay et al.^5^ reported three cases of anomalous single coronary artery detected incidentally during routine coronary angiography.

The SCA anomaly is mostly asymptomatic. However, some cases can result in severe life-threatening events such as myocardial ischemia, arrhythmia, and acute myocardial infarction.^1,2^ Recurrent chest pain without atherosclerosis in patients with SCA must be evaluated by computed tomography or pulmonary catheterization to determine the course of the artery.^6^

SCA is usually diagnosed incidentally during coronary angiographies or on postmortem evaluations. Multislice computed tomography is more effective than coronary angiography in determining coronary anomalies.^6,7^

The treatment strategy for single coronary artery has yet to be defined. Coronary artery bypass surgery might be useful in patients with anomalous coronary artery coursing between the aorta and main pulmonary artery or/and patients with atherosclerosis may benefit from revascularization procedures.
